# Case Report: A patient with spontaneous spinal epidural hematoma misdiagnosed as ischemic stroke recovered after delayed surgery

**DOI:** 10.3389/fmed.2026.1802695

**Published:** 2026-04-14

**Authors:** Jianli He, Qin Wang, Linna Jiao, Haibo Su, Liang Jin

**Affiliations:** 1 Xiaolan Clinical Institute of Shantou University Medical College, Zhongshan, Guangdong, China; 2 Department of Neurology, Xiaolan People’s Hospital of Zhongshan (The Fifth People’s Hospital of Zhongshan), Zhongshan, Guangdong, China; 3 Department of Anesthesiology, Xiaolan People’s Hospital of Zhongshan (The Fifth People’s Hospital of Zhongshan), Zhongshan, Guangdong, China

**Keywords:** delayed surgery, ischemic stroke, misdiagnosis, spontaneous spinal epidural hematoma, thrombolysis

## Abstract

Spontaneous spinal epidural hematoma (SSEH) manifesting acute hemiparesis is a rare stroke mimic that carries a high risk of mismanagement. We present the case of a 52-year-old man who presented with acute right hemiparesis and neck pain, initially diagnosed as an acute ischemic stroke (AIS) and treated with intravenous thrombolysis using tissue plasminogen activator (rt-PA). Persistent neck pain and subsequent urinary retention prompted re-evaluation of the initial cranial computed tomography angiography (CTA), which revealed an epidural hematoma. After limited improvement under 1 week of conservative management, he underwent surgical evacuation on day 12 post-onset, and achieved complete neurological recovery. This case underscores the importance of recognizing neck pain and bladder dysfunction as indicators of spinal pathology in the context of hyperacute stroke. It also demonstrates that delayed surgical intervention may still be effective in carefully selected SSEH patients with slow clinical progression and stable neurological status.

## 1 Introduction

Spontaneous spinal epidural hematoma (SSEH) is a rare and critical neurological emergency, which is defined as an epidural hematoma that occurs without preceding trauma or surgical intervention. Although the classic presentation is well characterized, diagnostic challenges may arise in atypical cases. In particular, SSEH manifesting as isolated hemiparesis closely simulates AIS, which may lead to misdiagnosis and unintended thrombolytic treatment (1–3). Use of rt-PA in patients with unrecognized SSEH has been linked to higher risks of hematoma enlargement and devastating neurologic decline (4, 5). Although urgent surgical decompression is considered the primary effective management (6), the optimal timing of surgery remains unclear. We report a patient with cervical SSEH who was misdiagnosed with AIS and given intravenous thrombolysis without complications. Delayed surgery performed on day 12 lead to complete functional recovery. This case highlights cognitive biases and imaging interpretation pitfalls in hyperacute stroke assessment, emphasizes the combined value of neck pain and urinary retention in differentiating spinal from cerebral etiologies, and provides evidence that extended surgical windows may be feasible in selected SSEH patients.

## 2 Case description

A 52-year-old right-handed man with a history of ischemic stroke and hypertension presented with right hemiparesis and neck pain after a morning jog. He denied a history of taking antiplatelet medication, statins, or antihypertensives. There were no visual disturbances, non-fluent speech, changes in voice, or difficulty swallowing. He was transported to the emergency department 2 h and 46 min after the onset of symptoms. His blood pressure was recorded at 157/95 mmHg. An initial neurological examination revealed a slightly shallow right nasolabial groove. The right upper limb muscle strength was grade 0, and the right lower limb muscle strength was grade 2. The left upper and lower limb muscle strength was grade 5. Sensory function examinations were normal. The National Institutes of Health Stroke Scale score was 7 at the time of presentation. The emergency department physician ordered cranial computed tomography (CT) and brain computed tomography angiography (CTA). The brain CT revealed no signs of hemorrhage and the carotid CTA showed no arterial dissection or other vascular abnormalities. The patient was diagnosed with an acute ischemic stroke, and intravenous thrombolysis with rt-PA (0.9 mg/kg) was considered with his consent 3 h and 10 min after the onset of symptoms. His neurological deficit did not improve after thrombolysis. He continued to complain of neck pain, and he experienced acute urinary retention. Hyperreflexia and ankle clonus were observed in both lower limbs at the same time.

The patient’s initial presentation with acute hemiparesis, combined with a history of previous stroke, led to a working diagnosis of recurrent acute ischemic stroke. Although stroke caused by vertebral artery dissection (VAD) often presents with neck pain, no signs of arterial dissection were observed in the initial head and neck CTA, and the patient had no clinical manifestations of posterior circulation infarction, such as vertigo, ataxia, swallowing difficulty. Therefore, vertebral artery dissection can be ruled out. However, the presence of persistent, significant neck pain and the subsequent onset of urinary retention were atypical for a cerebral infarct, prompting an exploration of alternative diagnoses.

**Figure 1:**
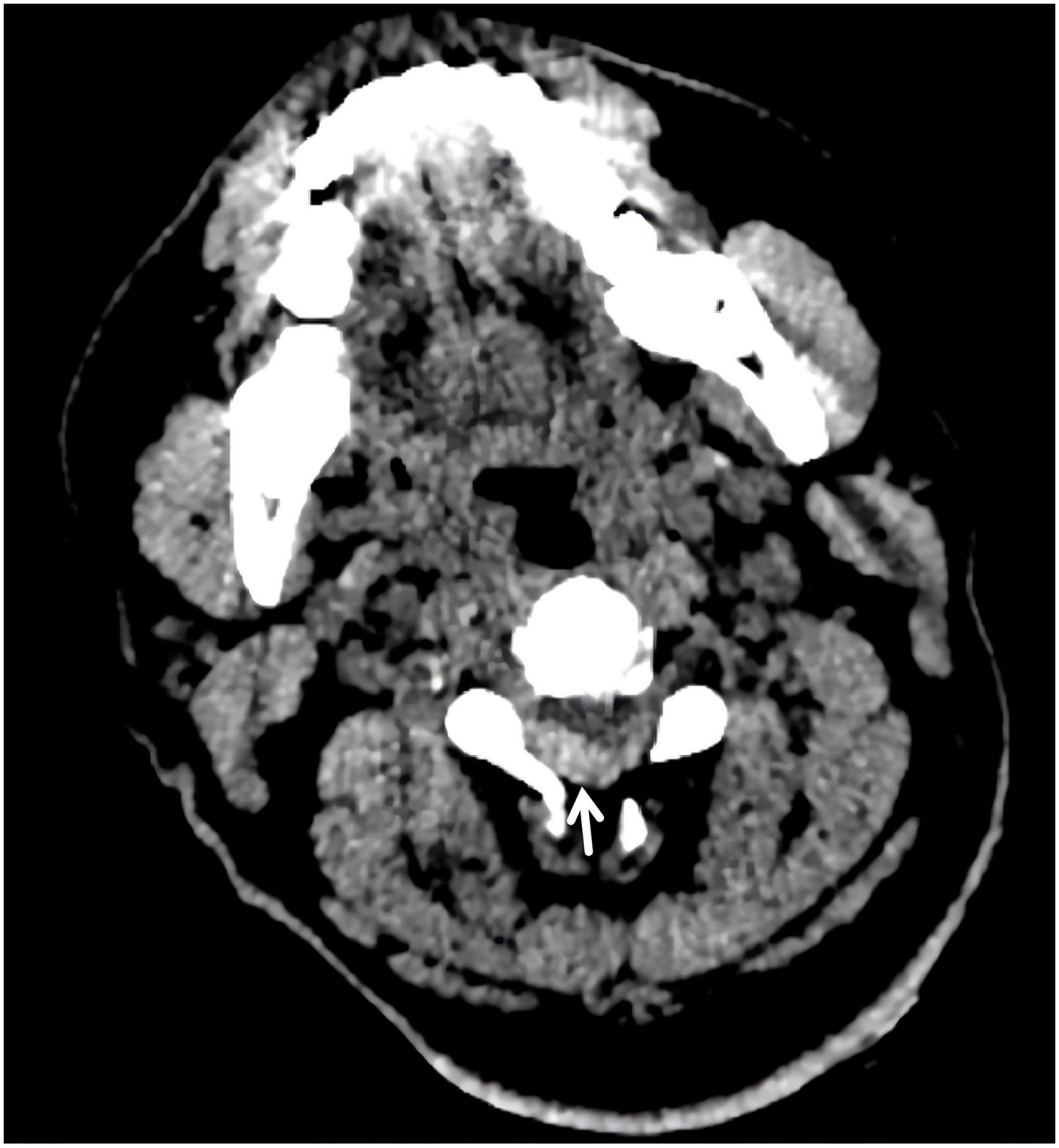
Cranial computed tomography angiography. An epidural lesion is visible within the cervical canal (white arrow). This finding was initially missed during hyperacute stroke assessment.

**Figure 2:**
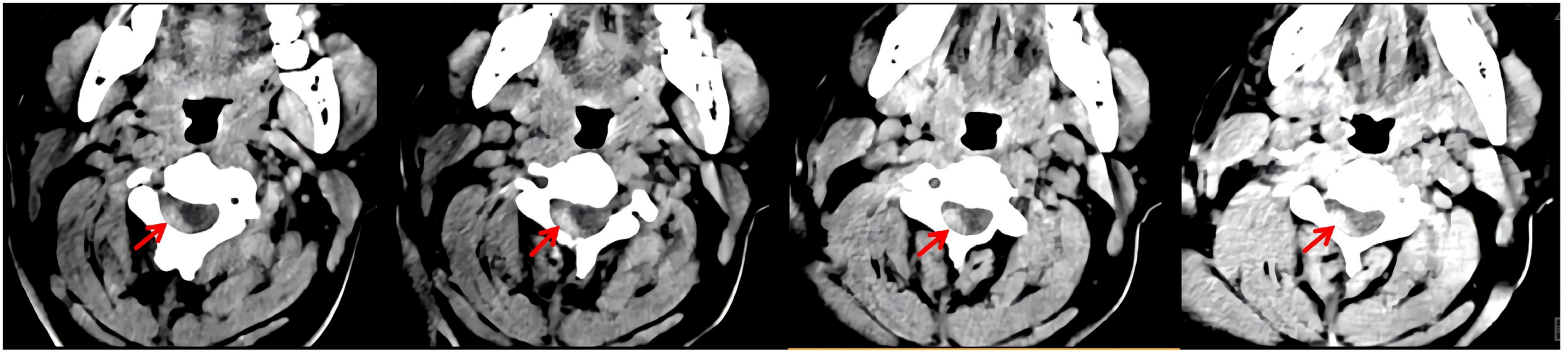
Cervical spine CT. A slightly hyperdense mass is observed in the right posterolateral epidural space (red arrows), consistent with an acute epidural hematoma.

**Figure 3:**
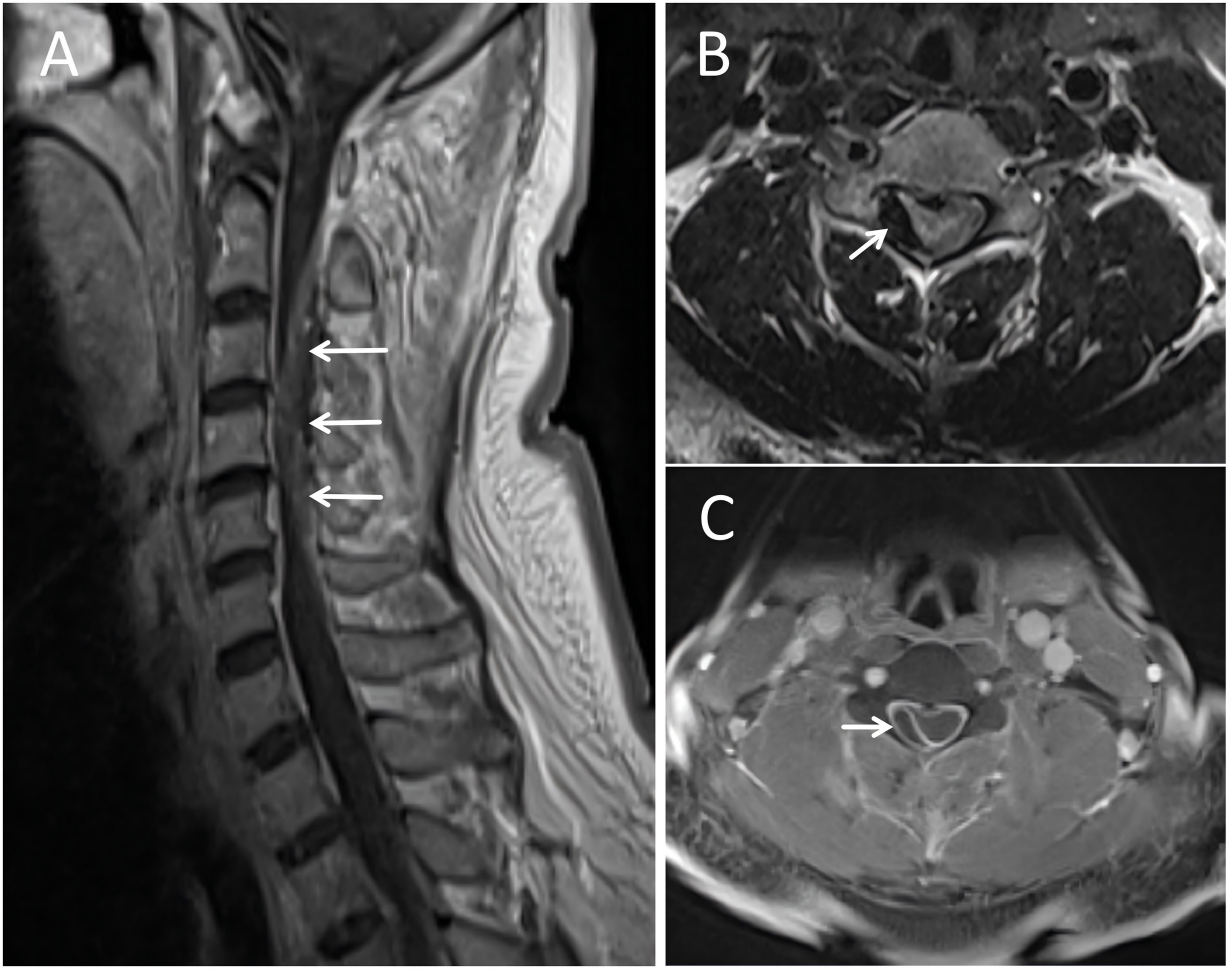
Cervical spine MRI. **(A)** Sagittal T1-weighted image demonstrates a slightly hyperintense signal within the mass in the dorsal epidural space from C2 to C6 (white arrows). **(B)** Axial T2-weighted image reveals a hypointense mass causing spinal cord compression (white arrow). **(C)** Axial contrast-enhanced T1-weighted image shows no enhancement of the mass (white arrow), confirming the absence of vascular malformation or neoplasm.

**Figure 4:**
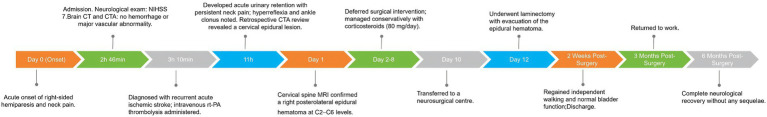
Timeline of clinical manifestations, diagnosis, and therapeutic interventions from symptom onset to six-month follow-up.

Laboratory tests including blood routine, blood biochemistry, coagulation function, and glycosylated hemoglobin, were within normal limits. Electrocardiogram (ECG) testing also returned normal findings. A retrospective review of the initial brain CTA, which included the cervical spine, revealed an epidural lesion in the cervical canal (Figure 1). A dedicated cervical spine CT confirmed a slightly hyperdense mass measuring approximately 0.5 cm x 8 cm located in the right posterolateral epidural space from C2 to C6 (Figure 2). Brain agnetic resonance imaging (MRI) showed no acute cerebral infarction, and subsequent MRI of the cervical spine revealed a non-enhancing extradural mass located along the right posterolateral aspect of the canal at the C2–C6 levels, consistent with an epidural hematoma that was causing spinal cord compression (Figure 3). As the patient and his family declined consent for urgent decompression surgery, he was treated with corticosteroids (80 mg daily). After 1 week, his muscle strength showed modest improvement (right upper limb: proximal was grade 2, distal was grade 3; right lower limb was grade 4), but urinary retention persisted. He was transferred to a neurosurgical center on day 10 and underwent a laminectomy with hematoma evacuation on day 12 post-onset. Two weeks post-surgery, he regained the ability to walk and was able to urinate spontaneously. He returned to work 3 months after the procedure. During a six-month follow-up after discharge, the patient remained in good health and exhibited no neurological deficits. The patient’s clinical presentation, diagnosis, and treatment measures are summarized in Figure 4.

## 3 Discussion

SSEH is a rare neurological emergency with an estimated annual incidence of 0.1 cases per 100,000 individuals (7). Most cases are observed aged between 40 and 60 (8). The underlying cause remains incompletely clarified; suggested risk factors include hypertension, diabetes mellitus, anticoagulant or antiplatelet therapy, hypothyroidism, anemia, deep diving, and pregnancy (9). The classic presentation of SSEH involves sudden spinal pain followed by rapidly progressive myelopathy, often manifesting as symmetric paraparesis or tetraparesis. However, presentation with isolated hemiparesis is uncommon and closely mimics AIS, creating a substantial diagnostic dilemma (2, 10).

The initial misdiagnosis in our patient can be attributed to cognitive biases. The presentation of acute hemiparesis in a patient with a prior history of ischemic stroke led the diagnostic team to favor cerebrovascular disease. This inclination was further exacerbated by the inherent pressure associated with the thrombolysis time window. However, several clinical features that deviated from a typical cerebral infarction should have a reevaluation of the initial diagnosis. The patient’s persistent neck pain is a significant sign that distinguishes SSEH from acute ischemic cerebrovascular diseases (5, 11). Moreover, the presence of acute urinary retention typically indicates spinal cord involvement rather than a cerebral hemispheric process (12). The neurological examination revealed hyperreflexia with knee and ankle clonus, which may have further raised suspicion.

Several cases of SSEH treated with intravenous thrombolysis have been reported, with most patients experiencing hematoma expansion and neurological deterioration (2, 6, 13–17). Our patient, in contrast, showed no clinical worsening after intravenous tissue plasminogen activator administration (IVT). We compared key clinical characteristics with those reported in the literature (Table 1). Several factors may explain the benign course. First, the hematoma was relatively small and confined to the right posterolateral epidural space, whereas cases that deteriorated after thrombolysis typically involved larger or circumferentially compressive hematoma (14, 15). Second, symptom progression was gradual in our patient, with stable neurological status after thrombolysis, in contrast to the rapid progression to tetraparesis reported in some deteriorating cases (2). The source of bleeding may also be relevant. Beatty and Winston’s classic hypothesis suggests that many SSEHs originate from the valveless, low-pressure epidural venous plexus, where bleeding is self-limited (18). Venous hematomas may form a stable clot more quickly than arterial bleeds, making them less susceptible to fibrinolysis.

**Table 1:** Characteristics of patient of SSEH that were misdiagnosed as stroke and treated with intravenous thrombolysis.

Author/year	Age/Sex	Spinal level	Hematoma size/location	Time from onset to tPA	Neurological change Post-tPA	Time to surgery	Outcome
Present case, 2026	52/M	C2-C6	0.5 cm × 8 cm, right posterolateral	3 h 10 min	No worsening	Day 12	Complete recovery
Szeto and Hui (16) (Patient 1)	61/F	C2-T1	Tiny, posterior	Not specified	No worsening	Conservative	Moderate disability
Szeto and Hui (16) (Patient 2)	58/M	C4-7	Not specified	Not specified	Worsened	Urgent	Good recovery
Son et al. (15)	63/M	C4-T2	Not specified, posterior	2 h 45 min	No improvement	2 h 45 min after tPA	Moderate disability
Teles et al. (6)	63/F	C3-C6	0.6 cm × 4 cm, right paramedian	3 h	worsened at 48 h	72 h	Moderate disability
Huang et al. (2)	54/F	C3	Not specified, right dorsal	Within 4.5 h	Worsened	12 h	Moderate disability
Morimoto et al. (14)	71/M	C4-C6	Not specified, left posterolateral	2 h	Worsened	24 h	Complete recovery
Patel et al. (13)	51/M	C3-C5	8 mm × 1.7 cm, right posterolateral	4.5 h	Worsened	48 h	Complete recovery
Rahangdale et al. (17)	67/M	Not specified	Not specified	Not specified	Worsened	Conservative	Good recovery

The radiological assessment of this case offers important lessons. The initial CTA of the head routinely includes the cervical spine. Retrospective review confirmed the hematoma was visible as a hyperdense epidural lesion in the cervical canal (Figure 1), particularly when on soft-tissue window settings (3), but it was initially overlooked. During hyperacute stroke evaluation, attention was focused on the intracranial vasculature, and the cervical spine soft tissues were not systematically examined. This reflects a common diagnostic pitfall: even when relevant findings are present within the scanned field, they may be missed if the reviewing physician does not actively survey all anatomical regions. We therefore emphasize that systematic review of the entire imaged field—including cervical spine soft-tissue windows—should be an integral part of the stroke imaging protocol.

Although some patients recover with conservative management (7), urgent surgical decompression (within 12 h of symptom onset) remains the gold standard for SSEH to maximize neurologic recovery (19–22). The duration of preoperative paralysis is inversely related to recovery prospects (23). Adverse prognostic factors include sphincter dysfunction, thoracic segment, severe neurological deficits on admission, the use of anticoagulant therapy, and a short progression interval (20). Most reported SSEH cases misdiagnosed as stroke underwent surgery within 72 h (3). Our patient underwent surgery on day 12 and achieved complete recovery. The slow progression and stable condition during initial conservative management may have allowed the spinal cord to adapt to compression, preserving recovery potential. However, this outcome should not be interpreted as evidence that delayed surgery is generally acceptable. Rather, it suggests that in selected patients with small hematomas, slow progression, and partial improvement with conservative care, the window for effective intervention may be broader. Early decompression remains the standard, and any decision to delay must be made cautiously on an individual basis.

Although SSEH mimicking stroke has been reported previously, this case offers three distinct and clinically meaningful lessons. First, Cognitive bias in acute stroke evaluation can lead to misdiagnosis despite the presence of atypical clinical features. Second, Imaging oversight on cranial CTA represents a common system pitfall that can be reduced by routine review of cervical soft-tissue windows. Third, delayed surgery can lead to complete neurologic recovery in carefully selected SSEH patients with slow progression and stable condition.

In conclusion, identifying SSEH in cases of acute hemiparesis poses a considerable diagnostic challenge for neurologists. When patients present with an acute onset of hemiparesis, especially when accompanied by neck pain or acute urinary retention, there is a high suspicion of SSEH. Appropriate imaging and the correct selection of window settings are crucial to prevent misdiagnosis.

## 4 Patient perspective

I suddenly could not move my right arm and leg while jogging, and the pain in my neck was severe. I thought it was another stroke. It was frightening to learn it was a spinal bleed, especially after receiving the “clot-busting” medication. I feel incredibly lucky that I did not get worse and that the surgery allowed me to fully recover and return to work.

## Data Availability

The original contributions presented in the study are included in the article/Supplementary material, further inquiries can be directed to the corresponding author.
