# Supplementary material for: Case Report: A patient with spontaneous spinal epidural hematoma misdiagnosed as ischemic stroke recovered after delayed surgery
Source: Front Med (Lausanne). 2026 Apr 14;13:1802695. doi: 10.3389/fmed.2026.1802695 (PMC13121364; doi:10.3389/fmed.2026.1802695)

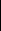

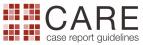

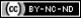

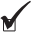
**CARE Checklist of information to include when writing a case report**

**Topic**

**Title**

**Key Words**

**Abstract**

**(no references)**

**Introduction**

**Patient Information**

**Clinical Findings**

**Timeline**

**Diagnostic Assessment**


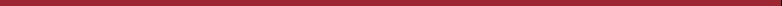


**Item Checklist item description Reported on Line**

**1** The diagnosis or intervention of primary focus followed by the words “case report” . . . . . . . . . . . . . . . . . . . . . . . . . . Title: “A patient with spontaneous epidural hematoma misdiagnosed as ischemic stroke recovered after delayed surgery：A case report”

**2** 2 to 5 key words that identify diagnoses or interventions in this case report, including "case report" . . . Keywords section

**3a** Introduction: What is unique about this case and what does it add to the scientific literature? . . . . . . . . . . . . . . . . Abstract

Section（ the first and last two sentences）

**3b** Main symptoms and/or important clinical findings . . . . . . . . . . . . . . . . . . . . . . . . . . . . . . . . . . . . . . . . . . . . . . . . . . . . . . . Abstract (the second and third sentences)

**3c** The main diagnoses, therapeutic interventions, and outcomes . . . . . . . . . . . . . . . . . . . . . . . .Abstract(the second to fourth sentences)

**3d** Conclusion—What is the main “take-away” lesson(s) from this case? . . . . . . . . . . . . . . . . . . . . . . . . . . . Abstract (last two sentences)

**4** One or two paragraphs summarizing why this case is unique (**may include references**) . . . . . . . . . . . . Introduction (entire paragraph)

**5a** De-identified patient specific information. . . . . . . . . . . . . . . . . . . . . . . . . . . . . . . . . . . . . . . . . . . . . . . . . . . . Case Description, Paragraph 1 (Age, sex, history)

**5b** Primary concerns and symptoms of the patient. . . . . . . . . . . . . . . . . . . . . . . . . . . . . . . . . . . . . . . . . . . . . . . . . . . . . Case Description, Paragraph 1 (hemiparesis and neck pain)

**5c** Medical, family, and psycho-social history including relevant genetic information . . . . . . . . . . . . . . . . . Case Description, Paragraph 1 (history of ischemic stroke and hypertension)

**5d** Relevant past interventions with outcomes . . . . . . . . . . . . . . . . . . . . . . . . . . . . . . . . . . . . . . . . . . . . . . . . . . . . . . . . Case Description, Paragraph 1 (denied medication history)

**6** Describe significant physical examination (PE) and important clinical findings. . . . . . . . . . . . . . . . . . . . . . . Case Description, Paragraph 1 (Neurological exam: muscle strength, NIHSS)

**7** Historical and current information from this episode of care organized as a timeline . . . . . . . . . . . . . . . Case Description (text); Figure 4 (Timeline)

**8a** Diagnostic testing (such as PE, laboratory testing, imaging, surveys). . . . . . . . . . . . . . . . . . . . . . . . . . . . . . . Case Description (Laboratory tests ,CT, CTA, MRI ,findings)

**8b** Diagnostic challenges (such as access to testing, financial, or cultural) . . . . . . . . . . . . . . . . . . . . . . . . . . . . . Discussion (first paragraph )

**Therapeutic Intervention**

**Follow-up and Outcomes**

**Discussion**

**Patient Perspective**

**Informed Consent**

**8c** Diagnosis(including other diagnoses considered) . .. . . . . . . . . . . . . . . . . . . . . .. . . . . . . . . . . . . . . . . . . .. . . . Case Description (diagnosis of SSEH); Page 2, Case Description (working diagnosis of stroke)

**8d** Prognosis (such as staging in oncology) where applicable . . . . . . . . . . . . . . . . . . . . . . . . . . . . Case Description (last paragraph): he regained the ability to walk,exhibited no neurological deficits at six-month follow-up

**9a** Types of therapeutic intervention (such as pharmacologic, surgical, preventive, self-care) . . . . . . . . . . . . . . . . . . . . Case Description: Thrombolysis, corticosteroids, surgical evacuation

**9b** Administration of therapeutic intervention (such as dosage, strength, duration) . . . . . . . . . . . . . . . . . . . . . . . . . . . . . Case Description (rt-PA at 3h10min, corticosteroids 80mg daily, surgery on day 12)

**9c** Changes in therapeutic intervention (with rationale) . . . . . . . . . . . . . . . . . . . . . . . . . . . . . . . . . . . . . . . . . . . . . . . . . . . . Page 2, Case Description: Change from conservative to surgical due to limited improvement.

**10a** Clinician and patient-assessed outcomes (if available) . . . . . . . . . . . . . . . . . . . . . . .. . . . . . . . . . . . . . . . . . . . . . . . . . . . Case Description: Muscle strength improvement, ability to walk, urination.

**10b** Important follow-up diagnostic and other test results . . . . . . . . . . . . . . . . . . . . . . . . . . . . . . . . . . . . . . . . . . . . . . . . . . . . Case Description: Six-month follow-up with no deficits **10c** Intervention adherence and tolerability (How was this assessed?) . . . . . . . . . . . . . . . . . . . . . . . . . . . . . . . Case Description: Tolerated thrombolysis without deterioration, initially declined consent for urgent decompression surgery

**10d** Adverse and unanticipated events . . . . . . . . . . . . . . . . . . . . . . . . . . . . . . . . . . . . . . . . . . . . . . . . . . . . . . . . . . . . . . . . . . . Case Description: No complications from thrombolysis.

**11a** A scientific discussion of the strengths AND limitations associated with this case report . . . . . . . . . . . . . . . . . . . . . . . Discussion (paragraphs on cognitive bias, imaging lessons, timing of surgery)

**11b** Discussion of the relevant medical literature **with references**. . . . . . . . . . . . . . . . . . . . . . . . . . . . . . . . . . . . . . . . . . Discussion (multiple paragraphs with references)

**11c** The scientific rationale for any conclusions (including assessment of possible causes) . . . . . . . . . . . . . . . . . . . . . . . . Discussion (paragraph 2,3,4)

**11d** The primary “take-away” lessons of this case report (without references) in a one paragraph conclusion . . . . . . . Discussion (last paragraph)

**12** The patient should share their perspective in one to two paragraphs on the treatment(s) they received . . . . . . . . Patient Perspective section

**13** Did the patient give informed consent? Please provide if requested . . . . . . . . . . . . . . . . . . . . . . . . . . . . . . . . . . . . . . **Yes** √ **No**
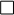

Supplement: Supplementary file 1 [file Supplementary_File_1.docx]
